# P-1321. Optimizing Survival: A Global Analysis of Antibiotic Regimens in Capnocytophaga canimorsus Infections

**DOI:** 10.1093/ofid/ofaf695.1509

**Published:** 2026-01-11

**Authors:** Hiyam Ghneim, Hashem Hajo Ebrahimi

**Affiliations:** University of Debrecen, Debrecen, Hajdu-Bihar, Hungary; University of Debrecen, Debrecen, Hajdu-Bihar, Hungary

## Abstract

**Background:**

*Capnocytophaga canimorsus* (CC) is a rare multi-drug resistant but rapidly fatal zoonotic infection associated with dog exposure, particularly affecting immunocompromised, asplenic, or elderly individuals. There are no FDA-approved drugs for CC infections. Empirical antibiotic therapy is diverse, with outcomes varying depending on the host’s baseline immune status and timing of intervention. This study investigates 64 CC cases to evaluate treatment patterns, demographic trends, and identify antibiotic regimens linked with optimal recovery.

**Table 1:**
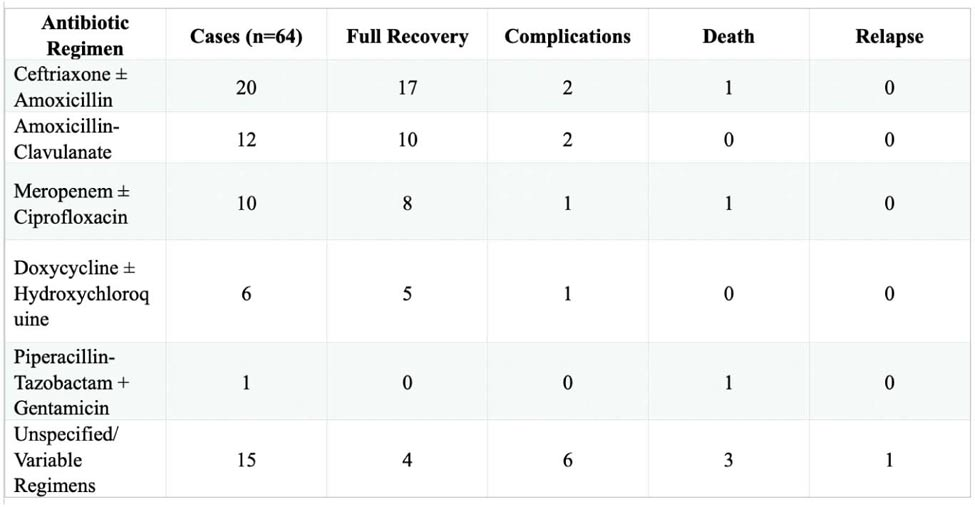
Antibiotic Regimens and Clinical Outcomes of Capnocytophaga canimorsus

**Figure 1:**
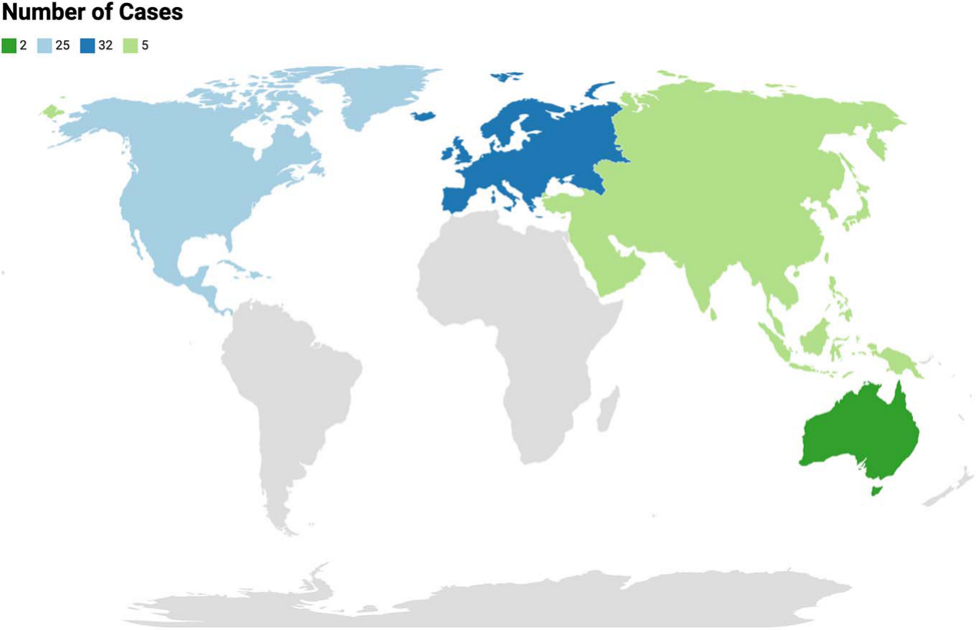
Geographic Distribution of Capnocytophaga canimorsus Cases This table summarizes the antibiotic therapies administered across 64 documented cases of Capnocytophaga canimorsus infection, alongside corresponding patient outcomes categorized as full recovery, complications, death, or relapse. Regimens involving ceftriaxone ± amoxicillin and amoxicillin-clavulanate were most frequently used and associated with the highest rates of full recovery. In contrast, the use of piperacillin-tazobactam plus gentamicin was linked to mortality. Patients treated with unspecified or variable regimens experienced the greatest incidence of poor outcomes, highlighting the need for early empiric coverage with high-efficacy agents in high-risk populations.

**Methods:**

64 *CC* cases published between 2010 and 2024 via PubMed and Embase were identified using relevant MeSH terms, Entree terms, and keywords. Rayyan.ai was used to screen articles following a PRISMA protocol. 64 cases met criteria and were manually reviewed. Demographics, immune status, geographic region, antibiotic combinations, and treatment outcomes were extracted and analyzed.

**Figure 2:**
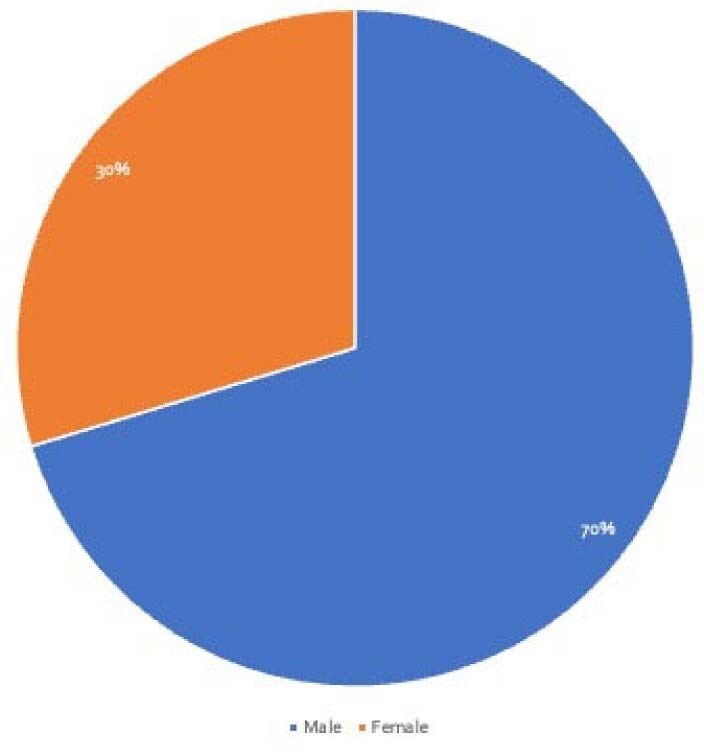
Gender Distribution of Patients with Capnocytophaga canimorsus Infection

**Figure 3:**
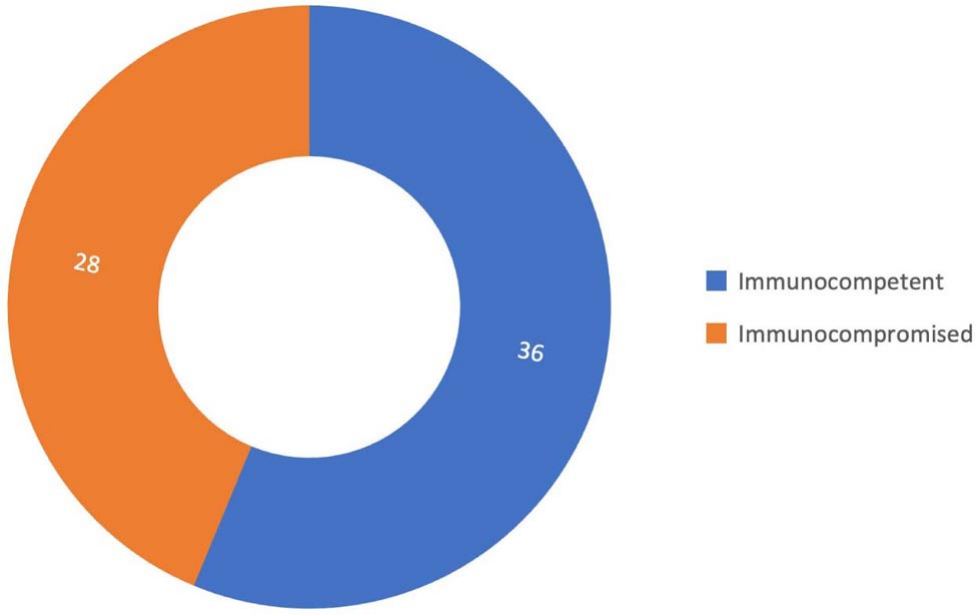
Immune System Status of Patients with Capnocytophaga canimorsus Infection

**Results:**

44/64 patients achieved full recovery with the most frequently used and effective regimen being ceftriaxone with amoxicillin at 85% recovery rate followed by amoxicillin-clavulanate and meropenem-based regimens (Table 1). Doxycycline and hydroxychloroquine was linked to favorable outcomes in beta-lactam-intolerant patients. In contrast, the only documented use of piperacillin-tazobactam combined with gentamicin resulted in death. Most cases originated from Western Europe at 30/64 cases and North America at 25/64 cases (Figure 1). 45/64 cases were males (Figure 2), and 28/64 were immunocompromised (Figure 3). These findings underscore the critical impact of early, targeted antibiotic selection in improving survival in *CC* infections.

**Conclusion:**

*CC* infection demonstrates a clear predilection for immunocompromised and male patients, with the highest incidence in Western Europe and North America. The antibiotic combination of ceftriaxone ± amoxicillin was associated with the best overall outcome, followed by amoxicillin-clavulanate and meropenem ± ciprofloxacin in severe cases. The use of piperacillin-tazobactam with aminoglycosides was linked to poor outcomes. This analysis highlights the need for early empiric beta-lactam therapy in high-risk hosts and supports the development of standardized treatment protocols.

**Disclosures:**

All Authors: No reported disclosures

